# DACH1 suppresses epithelial to mesenchymal transition (EMT) through Notch1 pathway and reverses progestin resistance in endometrial carcinoma

**DOI:** 10.1002/cam4.2317

**Published:** 2019-06-18

**Authors:** Qing Zhou, Wenzhi Li, Deshui Kong, Zhiming Liu, Zhengzheng Shi, Xiaohong Ma, Yongmei Li, Jie Jiang

**Affiliations:** ^1^ Department of Obstetrics and Gynecology Qi Lu Hospital, Shandong University Jinan Shandong China; ^2^ Department of Obstetrics and Gynecology The First Affiliated Hospital of Wenzhou Medical University Wenzhou Zhejiang China

**Keywords:** bioinformatics, DACH1, EMT, endometrial carcinoma, progestin resistance

## Abstract

Progestin resistance limits the effectiveness of progestin therapy in endometrial carcinoma for patients who desire to preserve fertility. To investigate the molecular mechanism of progestin resistance in endometrial carcinoma, we performed microarray analysis among Ishikawa and progestin resistant cell IshikawaPR cells. We found that epithelial to mesenchymal transition (EMT) was involved in progestin resistance and dachshund family transcription factor 1 (DACH1) is positively correlated with progesterone receptor (PGR). Knockdown of DACH1 in Ishikawa cell promoted proliferation, metastasis ability, and resistance to progestin. Conversely, overexpression of DACH1 in IshikawaPR cell rendered more sensitive to progestin treatment. Xenograft model assay also had similar results. In addition, our data showed that DACH1 overexpression inhibited EMT and decreased c‐Jun, Notch1 and Hes1expression. Our study demonstrated for the first time that EMT is involved in progestin resistance of EC. The response to progestin could be reserved by DACH1 suppressed EMT through Notch1 pathway via c‐Jun.

## 1 INTRODUCTION

Endometrial carcinoma (EC) is the most common gynecologic malignancy in developed countries. Hysterectomy is not an ideal treatment choice for patients who desire to preserve fertility or patients with server comorbidities that are not a suitable candidate for operation.^1^, ^2^ Synthetic progestin including medroxyprogesterone acetate (MPA) has been used as a conservative treatment therapy for a long time.^3^, ^4^ However, approximately 30% of early‐stage 1A EC patients fail to respond to progestin at presentation.^5^, ^6^ Response rate for advanced patients is only 20%‐40%.^7^ Although 70% of patients initially respond to progestin, 57% of them would recurrent and develop resistance.^6^ In conclusion, progestin resistance limits the effectiveness of progestin therapy. However, the precise molecular mechanism involved is poorly understood.

To explain the mechanism of MPA resistance, we previously developed a stable MPA resistant cell^8^ and performed microarray analysis to identify DEGs among Ishikawa and IshikawaPR cells. Progestin therapy is generally used for well‐differentiated endometrial cancer, and Ishikawa cell line was originally derived from a well‐differentiated adenocarcinoma of a 39‐year‐old woman in 1985,^9^ so we selected Ishikawa cell to developed progestin resistant cell line, because it is widely accepted that the presence of progesterone receptor (PR) is a prerequisite for progestin response.^10^ We firstly identified PGR correlated genes in EC and found the overlap genes with DEGs. Fifteen genes were selected and the role of DACH1 in progestin resistance of EC has become our interest.

DACH1 is a well‐conserved nuclear protein related to the Sno/Ski family of co‐repressors.^11^ Mounting evidence from recent studies show that DACH1 expression altered in many hormone‐responsive cancer (ovary, breast, prostate)^12^, ^13^, ^14^ and DACH1 could regulate hormone receptor signaling.^12^, ^14^ In breast cancer, associating with estrogen receptor (ER), DACH1 could inhibit estradiol‐induced DNA synthesis and cellular proliferation.^12^ We previously found that the DACH1 expression decreased in endometrial cancer.^15^ It indicated that DACH1 might be a tumor suppressor in EC. However, the specific role of DACH1 in progestin resistance of EC is not understood.

We also found that extracellular environment‐related Gene Oncology (GO) terms were significantly enriched in DEGs, and EMT marker was dramatically changed in IshikawaPR cell. It is well‐known that chemoresistance is frequently accompanied by EMT in diverse cancers. Recently, DACH1 has been shown to act as a negative regulator of EMT in breast cancer.^16^ Whether DACH1 could inhibit EMT and reverse progestin resistance in EC is required to research.

## 2 MATERIALS AND METHODS

### 2.1 Cell culture

Ishikawa cell was purchased from Shanghai Zhong Qiao Xin Zhou Biotechnology Co., Ltd. MPA resistant cell named IshikawaPR was established as we previously described.^8^ Ishikawa and IshikawaPR cells were routinely grown in RPMI 1640 (Hyclone, USA) containing 10% fetal bovine serum (FBS) at 3°C in a 5% CO_2_ humidified atmosphere. IshikawaPR cell was routinely cultured in 10 μmol L^−1^ MPA (Sigma‐Aldrich, St. Louis, MO, USA) to maintain resistance.

### 2.2 Microarray and bioinformatics analysis

We performed microarray analysis to identify DEGs among triplicate samples of parental Ishikawa and IshikawaPR cells. The raw data have been submitted to GEO (Series GSE121367 https://www.ncbi.nlm.nih.gov/geo/query/acc.cgi?&acc=GSE121367). GO enrichment analysis was performed using DAVID (https://david.ncifcrf.gov/). GO:0001837 (epithelial to mesenchymal transition)‐related genes list was downloaded from AmiGo 2 (http://amigo.geneontology.org/amigo/landing). Heat map was performed using Morpheus (https://software.broadinstitute.org/morpheus/). PGR coexpressed genes were identified with 507 EC tissues from cBioPortal (www.cbioportal.org). We used GSE17025, deposited by Day RS et al,^17^ to validate DACH1 expression profile and correlation between DACH1 and PGR in EC.

### 2.3 Real‐time quantitative reverse transcription PCR

The total RNA was extracted using TRIzol reagent (Invitrogen, Carlsbad, CA), and total RNA (3 μg) was reverse transcribed using M‐MLV reverse transcriptase (Cat no.C28025‐011, Invitrogen, China). Then, RNA expression was quantified using an ABI Prism 7500 Sequence Detection System (Applied Biosystems, USA) with SYBR Green Master Mix (Takara, Japan) in a 10‐μL reaction mixture. The primers were synthesized by Sangon Biotech Corporation and shown in Table S1.

### 2.4 Western blotting

Cell protein (30‐50 mg) was separated by 10%‐12% SDS‐PAGE gel, followed by electro‐blotted onto a polyvinylidene difluoride membranes (Millipore, Billerica, MA, USA). After blocking for 2 hours, membranes were incubated at 4°C overnight with the following primary antibodies: DACH1 (ab226176), E‐cadherin(ab76055), N‐cadherin(ab18203), VIM(ab92547), Hes1(ab221788)( Abcam, Cambridge, MA), c‐jun (#9165), p‐c‐jun(#2361), β‐catenin(#4970), cyclin D1(#2978), Caspase‐3 (#9665), cleaved‐caspase‐3(#9664), Notch1(#3608), and β‐actin(#4970) (Cell Signaling Technology, Beverly, MA). Then, membranes were incubated with secondary antibodies goat anti‐mouse IgG H&L (ab150113), anti‐rabbit IgG H&L(ab150080) for 1 hours. Blots were visualized using Immobilon^®^ Western Horseradish peroxidase substrate (Millipore, Billerica, MA, USA).

### 2.5 MTT assay

3‐(4, 5‐dimethylthiazol‐2‐yl)‐2, 5‐diphenyltetrazolium bromide (MTT) was applied to investigate cell proliferation and drug resistance. 0.1 × 10^4^ cells were seeded into a 96‐well plate and cultured overnight. Then, the original culture medium was replaced with fresh medium with indicated concentration of MPA. Then, 10‐μL MTT (5 mg mL^−1^ in PBS) was added in each well at 37°C for 4 hour. The formazan crystals were dissolved in 150 μL dimethylsulfoxide (Sigma‐Aldrich, St Louis, MO, USA). The absorbance of wells was detected at 490 nm wavelength.

### 2.6 EDU incorporation assay

5‐ethynyl‐20‐deoxyuridine (EdU) incorporation assay kit (Ribobio, Guangzhou, China) was used according to manufacturer's instruction. 1× 10^4^ cells per well were seeded in a 96‐well plate and incubated overnight and then replaced by 0, 30, 60 μmol L^−1^ MPA, respectively. After 48‐h incubation, 50‐μmol L^−1^ EdU was added for 4 hour before fixation, permeabilization, and staining. Finally, cell nuclei were stained with 1 × Hoechst nuclear dye for 30 min and then detected by fluorescence microscopy.

### 2.7 Analysis of cell apoptosis by flow cytomentry (FCM)

The apoptosis was detected by BD Pharmingen FITC annexin V Apoptosis Detection Kit I (BD Biosciences). Following the manufacturer's instruction, cells were collected, stained, gently vortexed, and incubated for 15 min at room temperature before measuring by FCM (BD Bioscience, FACS Calibur). Data were analyzed using CellQuest Pro software.

### 2.8 Wound healing assay

20× 10^4^ cells per well were seeded in 24‐well plates and incubated overnight and then replacing with fresh medium containing 0, 30, 60 μmol L^−1^ MPA, respectively. Forty‐eight hours later, wounds were scratched using 10‐μL pipette tips. Cells were cultured until they reached confluence and photographed at 0, 24, 48, 72, 96 hours.

### 2.9 Cell migration assay

60× 10^4 ^cells were seeded in a 60‐mm culture dish overnight and administration with different concentration of MPA for 24 hour. 6× 10^4^ cells in 100‐μL serum‐free medium were seeded into the upper Transwell chamber (catalog number 3422; Corning Life Sciences, Corning, NY, USA). A known value of 700 μL medium with 10% FBS was added to the lower chamber. The chambers were removed 24 hour after incubation at 37°C. Cells that penetrate through the membrane were fixed with 95% ethanol for 15 min and stained by 0.1% Crystal Violet for 30 min. The number of migrating cells was counted using the up‐right fluorescence microscope in five random views (200×).

### 2.10 Lentivirus packaging and infection

The DACH1 overexpression and one short hairpin RNA (shRNA) lentivirus were purchased from Genechem Co. Ltd (Shanghai, China). For DACH1 knockdown, shRNA targeting the sequence of 5’GATGGGCTTATCACCAAAT3’ and the control sequence 5ʹTTCTCCGAACGTGTCACGT3’ was subcloned into the GV112 vector. Full‐length DNA of human DACH1 (NM_080759) was cloned into the vector GV492. Ishikawa and IshikawaPR cells were infected with the lentivirals according to manufacturer's protocol. Stable transfectants were selected and cultured in medium containing 3 μg mL^−1^ puromycin for 5 days.

### 2.11 Xenograft model and drug resistance assay in vivo

The BALB/c nude mice (female, aged 6 weeks, 17.3 ± 3.6), purchased from Beijing Vital River Laboratory Animal Technology Co., Ltd, were housed in SPF breeding units. Each nude mouse received a bilateral subcutaneous inoculating of 8×10^6^ cells suspension. Control cells were injected into the left axilla, and DACH1 overexpression and knockdown cells were transplanted into the right axilla. The transplanted tumors had developed 14 days after injection. Twenty successful mouse models were randomly divided into 4 groups, shRNA N.S (normal saline), shRNA MPA treatment, pCMV N.S, and pCMV MPA treatment group. MPA (100 mg kg^−1 ^d^−1^) and equal volume N.S of were intraperitoneally injected into nude mice every 2 days for 9 times. All mice were killed by anesthetic overdose on day 32. The length (mm) and width (mm) of the tumor were measured. Tumor volume was calculated: tumor volume = width^2^ × length/2.

### 2.12 Statistical analysis

The data were analyzed using GraphPad 7.0 software and expressed as mean ± SD. Statistical significance was performed by Student's *t* tests, one‐way ANOVA, and two‐way ANOVA analysis. Correlation analysis was performed with SPSS 22.0. Differences of *P* < 0.05 were considered significant for all statistical tests.

## 3 RESULTS

### 3.1 Epithelial to mesenchymal transition (EMT) is involved in progestin resistance

**Figure 1:**
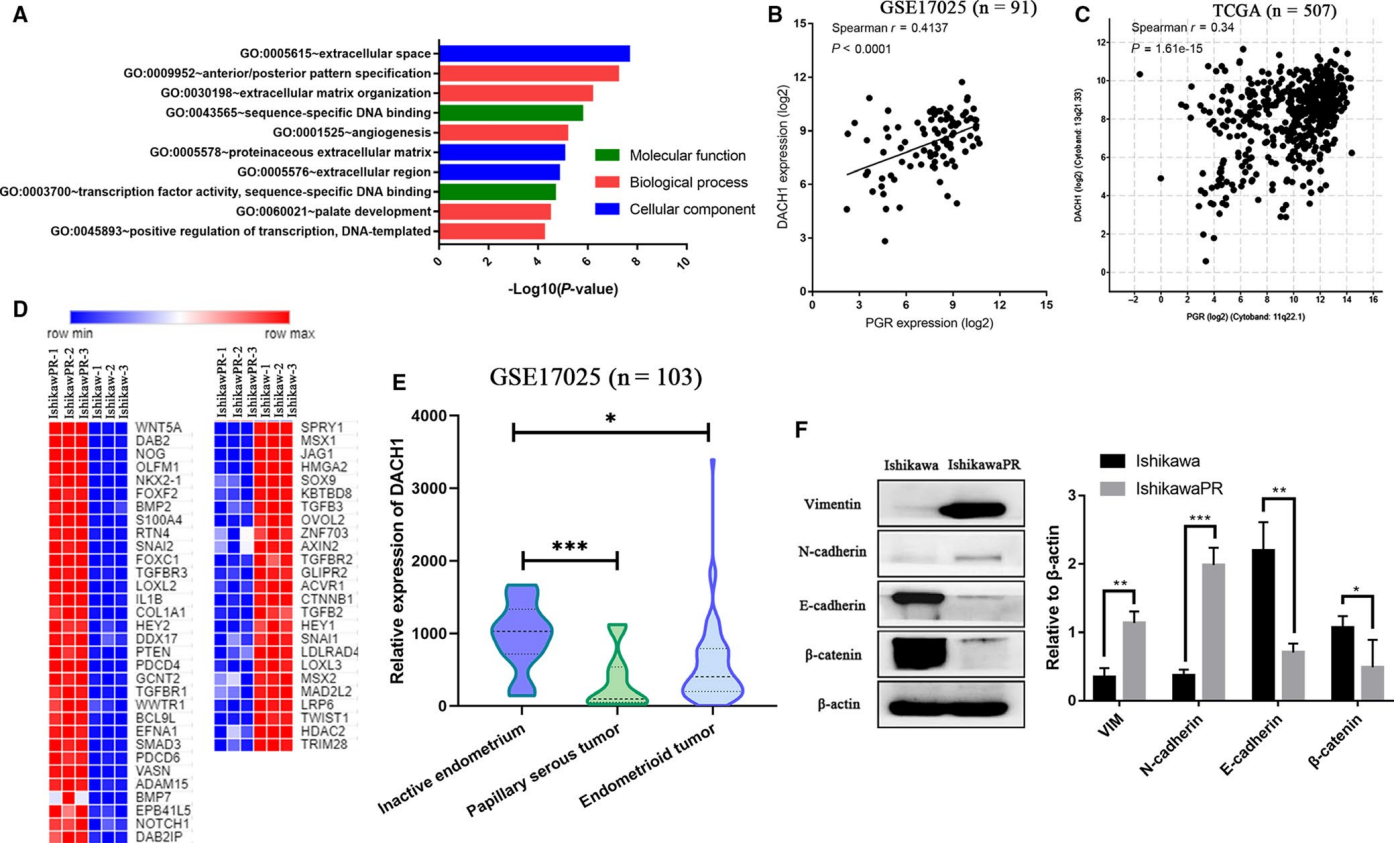
Epithelial to mesenchymal transition (EMT) is involved in progestin resistance and DACH1 expression is positively correlated with PGR. (A) TOP 10 GO enrichment terms of DEGs in order of *P*‐value. (B) Correlation analysis between DACH1 and PGR in EC tissues of GSE17025 (n = 91). (C) Correlation analysis between DACH1 and PGR expression was in EC of TCGA dataset (n = 507). (D) heatmap of 57 genes that are annotated to EMT (GO:0001837) term and dramatically changed in IshikawaPR cell. Red represents upregulated and blue represents downregulated in IshikawaPR cell. (E) Violin plot showed the expression profile of DACH1 in different endometrioid tissues of GSE17025 (n = 103). (F) EMT markers expression in IshikawaPR and Ishikawa cells

We developed a MPA stable resistant EC cell line IshikawaPR.^8^ We performed microarray analysis, and the raw data have been uploaded to GEO database (GSE121367). A total of 821 DEGs were extracted, including 453 upregulated and 368 downregulated genes in IshikawaPR cell compared with Ishikawa cell. We performed GO enrichment analysis and found that 4 of TOP 10 GO enriched terms were related to extracellular matrix (Figure 1A). As shown in Figure 1C, 57 genes annotated to EMT (GO:0001837) were dramatically changed in IshikawaPR cell.

Concomitant with the MPA resistance characteristic, IshikawaPR cell underwent significant morphological changes (Figure S1A). As shown in Figure S1B and C, enhanced metastatic characteristics were detected in IshikawaPR cell using wound healing and Transwell assay. Epithelial marker E‐cadherin and β‐catenin were downregulated, and mesenchymal markers Vimentin and N‐cadherin were upregulated in IshikawaPR cell compared with Ishikawa cell. (Figure 1F).

### 3.2 DACH1 is downregulated in IshikawaPR cell and positively correlated with PGR

Constant stimulation of progesterone reduced the expression of PGR and developed drug resistance.^10^ We performed PGR coexpression analysis based on 507 EC tissues. A total of 859 PGR coexpressed genes were identified. Then, we intersected two gene lists (PGR coexpressed genes and DEGs) and validated the genes in GEO database GSE17025. Finally, 15 genes that were correlated with PG R and altered in IshikawaPR cell were selected. By coincidence, we previously researched on the altered DACH1 in EC within 126 endometrium specimens. We found that DACH1 expression in EC decreased than normal endometrium.^15^ So we speculated that MPA resistance was modulated by DACH1. Firstly, we analyzed DACH1 expression in GSE17025. Consistent with our previous study, DACH1 was downregulated in papillary serous tumor and endometrioid tumor (Figure 1E). A significantly positive correlation was found between DACH1 and PGR in TCGA datasets (n = 507) and GSE17025 (n = 91) (Figure 1B and 1). In addition, we treated Ishikawa cell with MPA at 0, 24, 48, 72 hours and as shown in Figure S1D, DACH1 clearly decreased after treatment with 15 μmol L^−1^ MPA for 72 hours.

### 3.3 Suppression of DACH1 promotes proliferation, migration, and induces MPA resistance

**Figure 2:**
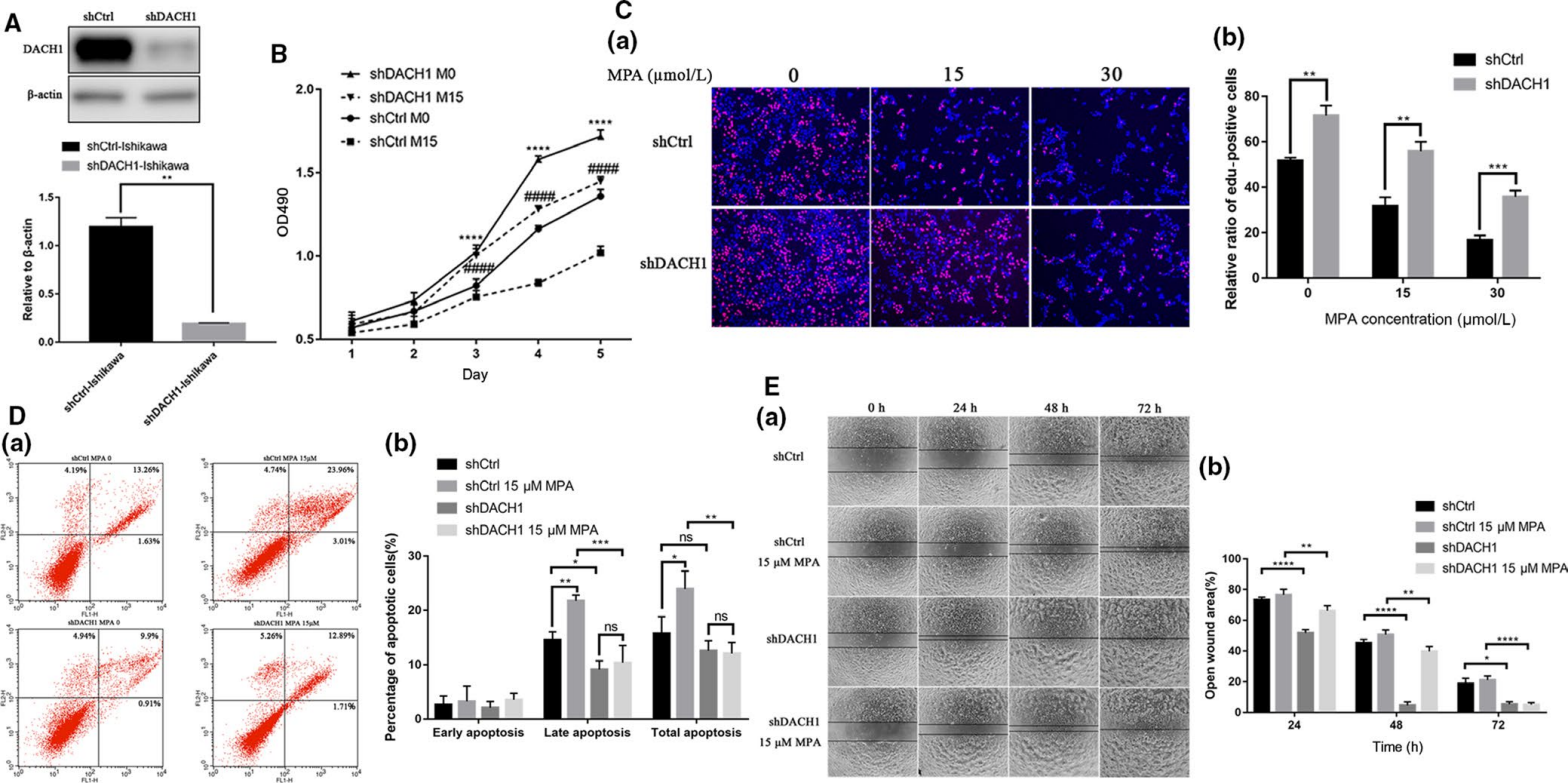
Suppression of DACH1 promotes proliferation, migration and induces MPA resistance. (A) Knockdown efficiency of DACH1 in Ishikawa cell. (B) Cell growth curve of shDACH1 and shCtrl Ishikawa cells at 0, 15 μmol L^−1^ MPA was examined by MTT assay at 1‐5 day. ^****^ *P* < 0.0001 shDACH1 cells treated without MPA vs shCtrl treated without MPA at the same day. ^####^ *P* < 0.0001 shDACH1 cells treated with 15 μmol L^−1^ MPA vs shCtrl treated with 15 μmol L^−1^ MPA at the same day. (C) EdU incorporation assay after incubation with 0, 15, 30 μmol L^−1^ MPA respectively for 48 hour. (D) Flow cytometry showed that apoptosis cells was decreased in shDACH1 Ishikawa cell than shCtrl with or without MPA incorporation. No obvious change in shDACH1 when treated with 15 μmol L^−1^ MPA. (E) Knockdown of DACH1 enhanced migration capacity of Ishikawa compared with shCtrl Ishikawa by wound healing assays

**Figure 3:**
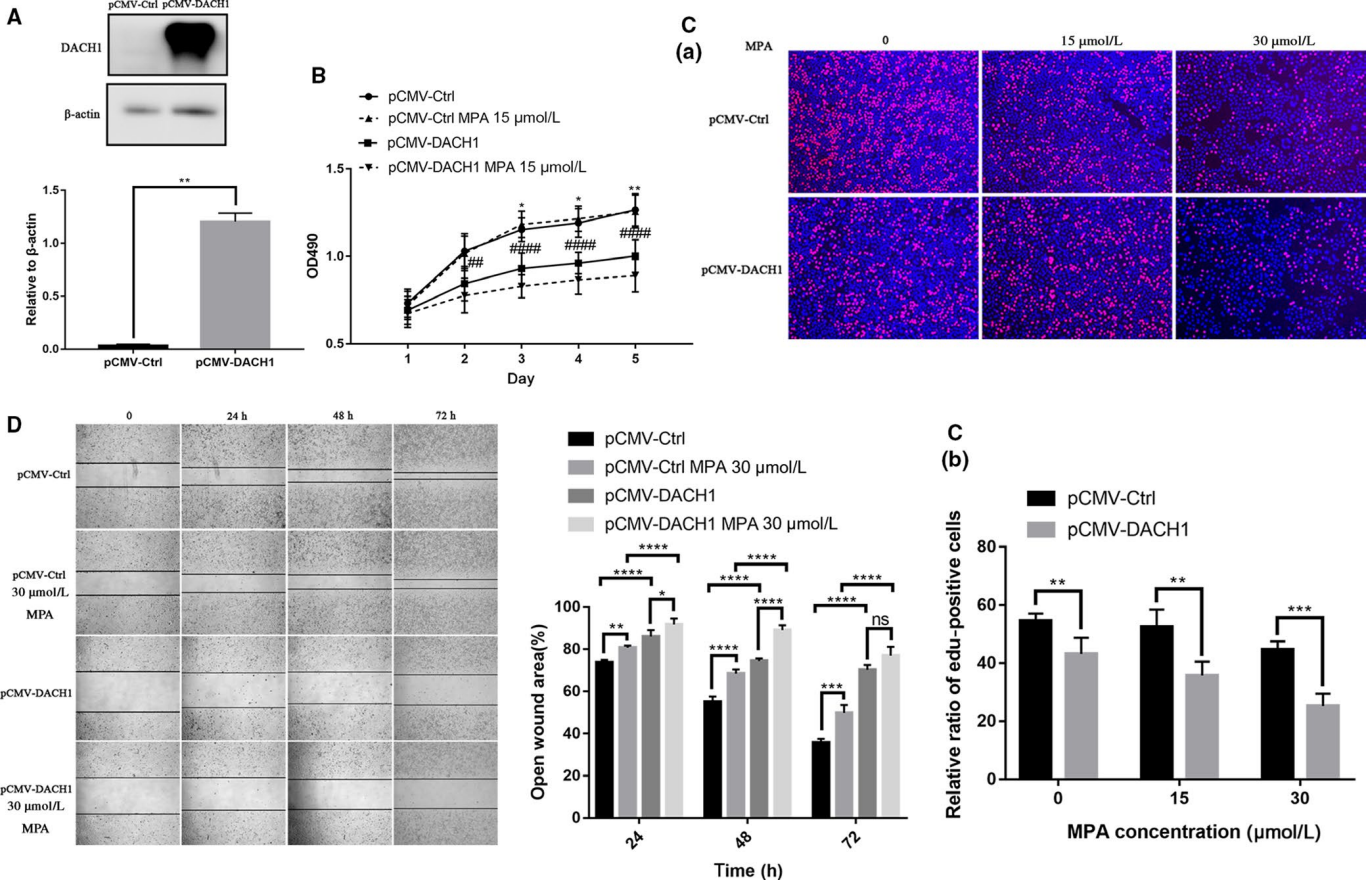
DACH1 overexpression suppresses proliferation, migration and reverse resistance to MPA. (A) Overexpression efficiency of DACH1 in IshikawaPR cell. (B) Cell growth curve showed a decrease in cell viability upon nondisclosure or exposure to 15 μmol L^−1^ MPA at 2‐5 day was much more pronounced in pCMVDACH1 IshikawaPR cell than Ctrl cell by MTT assay. * *P* < 0.05 pCMV‐Ctrl cultured without MPA vs pCMVDACH1 cultured without MPA. # *P* < 0.05 pCMV‐Ctrl treated with 15 μmol L^−1^ MPA vs pCMV‐DACH1 treated with 15 μmol L^−1^ MPA. (C) EdU incorporation assay showed the DNA synthesis is significantly decreased in pCMVDACH1 IshikawaPR cell than control group at 0, 15, 30 μmol L^−1^ MPA. (D) Wound healing assay showed reduced migration abilities in pCMV‐DACH1 IshikawaPR cell

As DACH1 expression was positively correlated with PGR expression, we hypothesized that DACH1 affects response to progestin. Firstly, we silenced DACH1 by transfecting Ishikawa cell with shRNA lentivirus. The efficiency was detected by Western blot (Figure 2A). The data showed that cell viability of shDACH1 Ishikawa cell significantly increased at 3‐5 days when treated with 0, 15 μmol L^−1^ MPA, respectively, compared with shCtrl cell by MTT assay (Figure 2B). MPA‐induced cell growth inhibition effect was blocked in shDACH1 Ishikawa cell when treated with 15 μmol L^−1^ MPA for 48 hour by EdU assay (*P* > 0.1) (Figure 3C). Moreover, flow cytometry showed that the percentage of apoptosis cells especially late apoptosis was decreased in shDACH1 Ishikawa cell than shCtrl with or without MPA incorporation, but there was no obvious change in apoptosis of shDACH1 when treated with 15 μmol L^−1^ MPA (Figure 2D). As shown in Figure 2E, knockdown of DACH1 enhanced migration capacity of Ishikawa compared with shCtrl Ishikawa by wound healing assay.

### 3.4 DACH1 overexpression suppresses proliferation, migration, and reverse resistance to MPA

Next, we established stably transfected pCMV‐DACH1 lentivirus IshikawaPR cell, and DACH1 expression significantly increased (Figure 3A). MTT assay showed the decrease in cell viability at 2‐5 day was much more pronounced in pCMV‐DACH1 IshikawaPR cell than Ctrl in 15 μmol L^−1^ MPA treatment (Figure 3B). We performed an EdU incorporation assay to visualize the response to MPA on DNA synthesis in IshikawaPR cell. DNA synthesis is significantly decreased in pCMV‐DACH1 IshikawaPR cell than control group at 0, 15, 30 μmol L^−1^ MPA (Figure 3C). The migration experiment showed reduced migration abilities in pCMV‐DACH1 IshikawaPR cell (Figure 3D). Taken together, these data supported that DACH1 plays an important role in proliferation, migration, and response to MPA of EC cell.

### 3.5 DACH1 influences EMT and NOTCh1 pathway via c‐JUN

**Figure 4:**
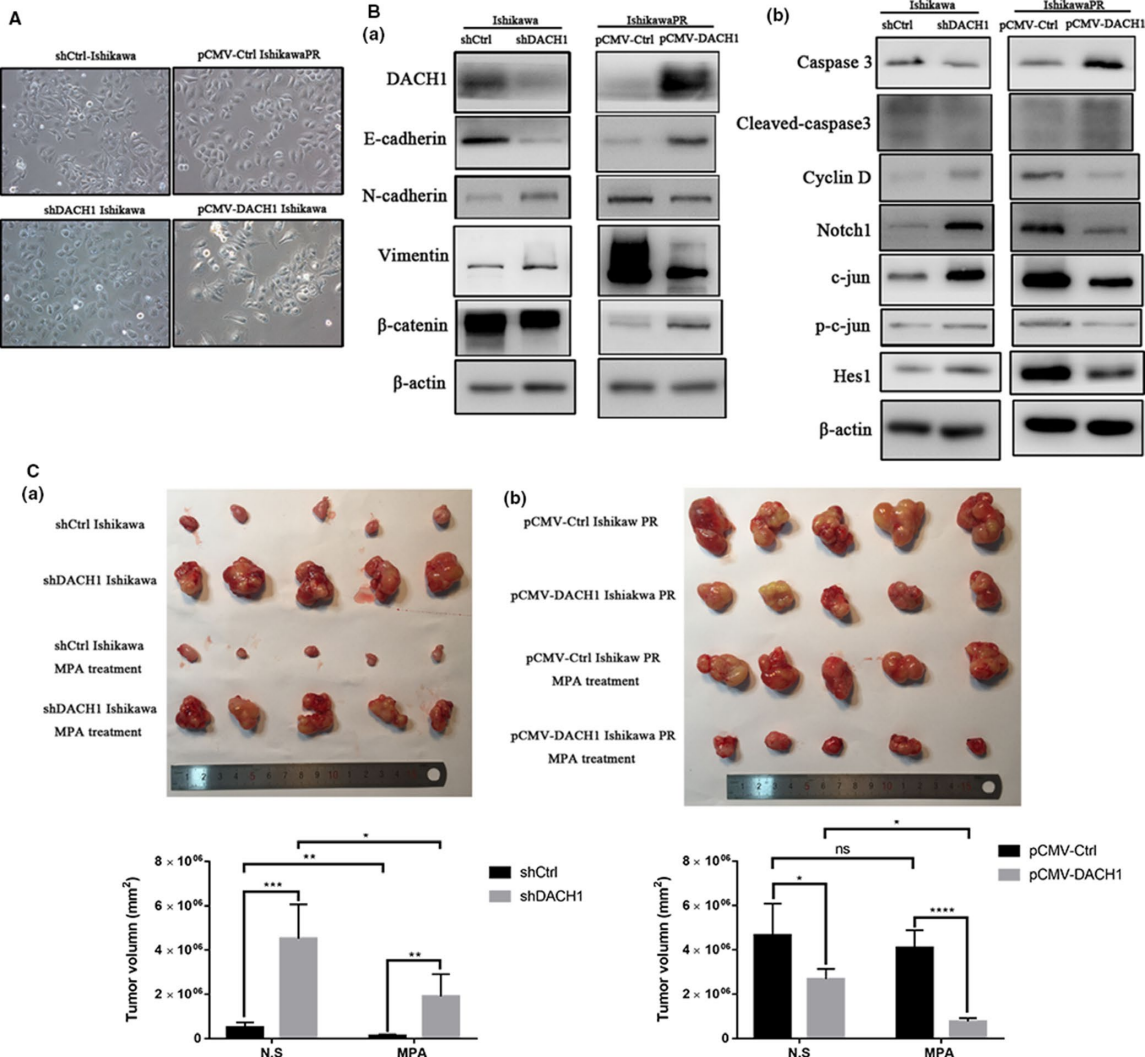
DACH1 influences expression of EMT‐related genes, Notch1 pathway, c‐Jun and tumor growth in vivo. (A) Cell morphology of stably knockdown and overexpressing DACH1 Ishikawa and IshikawaPR cell. (B) Effect of DACH1 knockdown and overexpression on EMT marker (E‐cadherin, N‐cadherin, Vimentin and β‐catenin), Notch1, Hes1, c‐Jun and p‐c‐Jun expression. (C) Knockdown and overexpression of DACH1 influence tumor growth and MPA resistance in vivo

To explore the molecular mechanism of DACH1 regulating the response to MPA in EC cell, EMT marker and Notch pathway proteins were detected by Western blot. As shown in Figure 4A, cell morphology of Ishikawa cell reverted to the mesenchymal in silencing of DACH1. An adverse morphological change was observed in pCMV‐DACH1 IshikawaPR cell. Accompanied with the morphological change, the expression of N‐cadherin and Vimentin was upregulated, and E‐cadherin and β‐catenin were downregulated in shDACH1 Ishikawa cell. In contrast, increased E‐cadherin and β‐catenin and decreased N‐cadherin and Vimentin were observed in IshikawaPR cell in DACH1 overexpression (Figure 4Ba). These results indicated that DACH1 depletion enhanced the mesenchymal transformation.

The results showed that DACH1 increased caspase 3 and Cleaved‐caspase 3, decreased Cyclin D, c‐Jun and p‐c‐Jun, Notch1, and its target gene Hes1. shDACH1 Ishikawa cell presented the opposite transformation of these proteins (Figure 4Bb). These results indicated that DACH1 can reverse MPA resistance and EMT through Notch1 pathway via c‐Jun.

### 3.6 DACH1 regulates tumor growth and MPA response in vivo

We established endometrial cancer xenograft model inoculating with Ishikawa and IshikawaPR cell expressing vector, shDACH1, or pCMV‐DACH1. After 14 days, tumors had developed and the growth rate is remarkably different. Then, MPA treatment (100 mg kg^−1 ^day^−1^) and equal volume of N.S were intraperitoneally injected into nude mice every 2 days for 9 times.

The results showed that there was an obvious difference in the response to MPA. Although there was an inhibition after MPA treatment in tumors with shDACH1 Ishikawa cell, the inhibition rate was lower than shCtrl group. Conversely, for tumors with pCMV‐Ctrl IshikawaPR, there is no significant inhibition effect to MPA treatment, but MPA significantly suppressed tumor growth in pCMV‐DACH1 group (Figure 4C). Taken together, these in vivo results indicate that knockdown of DACH1 promotes tumor growth of EC and MPA resistance.

## 4 DISCUSSION

Endometrial cancer is the most common gynecologic malignancy in developed countries. Progestin resistance is a major clinical problem that reduced the efficacy of progestin therapy. In order to understand the molecular mechanisms of acquired MPA resistance in EC, we previously established a stable MPA resistant Ishikawa cell line^8^ and we performed microarray analysis to identify the DEGs. The result showed that 821 DEGs were extracted. GO enrichment analysis revealed that extracellular environment and EMT were significantly changed in IshikawaPR cell. Numerous evidence have shown the vital role of EMT in chemoresistance of diverse cancer (breast cancer, bladder cancer, and pancreatic cancer).^18^, ^19^, ^20^, ^21^ Our results showed that epithelial markers E‐cadherin and β‐catenin were dramatically decreased, and mesenchymal markers Vimentin and N‐cadherin were increased in IshikawaPR cell. These findings are the first time to suggest that EMT is involved in acquired progestin resistance in EC. However, the molecular mechanism that EMT was regulated in EC remains unknown.

It is widely believed that the presence of progesterone receptor is the prerequisite of progestin response, and PR is a predictive marker for response to progestin.^22^ Continuous administration of progestin reduced the expression of PR^8^, ^10^ To explain the molecular mechanism of regulating PR, we performed PGR coexpressed correlation analysis and found the overlap genes with DEGs. Interestingly, 15 genes were identified and DACH1, which we previously verified the altered expression in EC, is one of them. Furthermore, consistent with correlation analysis and our previous results, DACH1 is lower expressed in EC tissues compared with normal endometrium and is positively correlated with PGR.

DACH1 is a highly conserved nuclear protein localizes to chromosome 13q21.^23^ Recent studies have demonstrated that DACH1 expression is altered in different types of hormone‐responsive cancers (breast, ovary, and prostate).^12^, ^13^, ^14^ The inhibition role of DACH1 in oncogene‐induced cellular proliferation and migration is verified in breast cancer cells and DACH1 regulates hormone‐dependent signaling.^12^, ^14^ Hence, we explored the functional studies of DACH1 in EC cell. The results showed that shDACH1 significantly promoted cell viability, migration capacity of Ishikawa cell, and increased resistance to progestin. Conversely, DACH1 overexpression suppressed proliferation, metastasis ability, and sensitized the IshikawaPR cell to progestin. In vivo assay was consistent with in vitro studies. Similar findings were also observed in gastric cancer that DACH1 was dramatically lower expressed in chemoresistant compared with chemosensitive tumors and could be an independent predictor for chemoresistance.^24^ Overall, these results suggest that DACH1 could suppress tumor progression and reverse acquired progestin resistance.

Studies have revealed that DACH1 inhibits SNAI1, and TGF‐β‐mediated EMT is involved in breast cancer,^16^, ^25^ which promoted us to explore the possibility that DACH1 regulates EMT in progestin resistance. In this study, we observed that N‐cadherin and Vimentin were upregulated, and E‐cadherin and β‐catenin were downregulated in shDACH1 Ishikawa cell. In contrast, DACH1 overexpression resulted in increased E‐cadherin and β‐catenin but decreased N‐cadherin and Vimentin in IshikawaPR cell. These results supported the role of DACH1 in regulation of EMT. As studies showed that DACH1 binds to c‐Jun and inhibits its function of contact‐independent growth in breast cancer cells,^13^, ^26^ and JNK/c‐Jun signaling pathway promoted cancer stem‐like cell (CSC) phenotype through Notch1 signaling in triple‐negative breast cancer (TNBC),^27^ we speculated that DACH1 might reverse EMT by suppressed Notch1 pathway via c‐Jun. Our data preliminarily validated this hypothesis that DACH1 overexpression decreased Cyclin D, c‐Jun and p‐c‐Jun, Notch1, and its target gene Hes1 expression. Knockdown of DACH1 presented opposite transformation of these proteins.

It has been verified that DACH1 could colocalized with ERα in breast cancer and AR in normal prostate.12,14 Whether DACH1 could directly bind with PR and its underlying mechanism remains elusive Understanding the molecular mechanism between DACH1 and PR involved in progestin resistance will be used to design better intervention strategies to predict and reverse progestin resistance of EC.
