# Supplementary material for: DACH1 suppresses epithelial to mesenchymal transition (EMT) through Notch1 pathway and reverses progestin resistance in endometrial carcinoma
Source: Cancer Med. 2019 Jun 18;8(9):4380–8. doi: 10.1002/cam4.2317 (PMC6675747; doi:10.1002/cam4.2317)
Supplement: Supplementary file 1 [file CAM4-8-4380-s001.docx]

**Supplementary Table 1**. The information of primers used in this study.

| **Gene name** | **Primer sequence** | |
| --- | --- | --- |
| DACH1 | Forward | CCATGAGCAACTATCATGCC |
|  | Reverse | TGTCCATGCCCAGTTAGAGA |
| N-cadherin | Forward | TCGCCATCCAGACCGACCCA |
|  | Reverse | TGAGGCGGGTGCTGAATTCCC |
| E-cadherin | Forward | GCCGCTGGCGTCTGTAGGAA |
|  | Reverse | TGACCACCGCTCTCCTCCGA |
| Vimentin | Forward | TGGATTCACTCCCTCTGGTTG |
|  | Reverse | CGTGATGCTGAGAAGTTTCGTT |
| Snail1 | Forward | TCGGAAGCCTAACTACAGCGA |
|  | Reverse | AGATGAGCATTGGCAGCGAG |
| Twist2 | Forward | GCGCAAGTGGAATTGGGATG |
|  | Reverse | CGGGTCTTCTGTCCGATGTC |
| β-actin | Forward | CATGTACGTTGCTATCCAGGC |
|  | Reverse | CTCCTTAATGTCACGCACGAT |

**
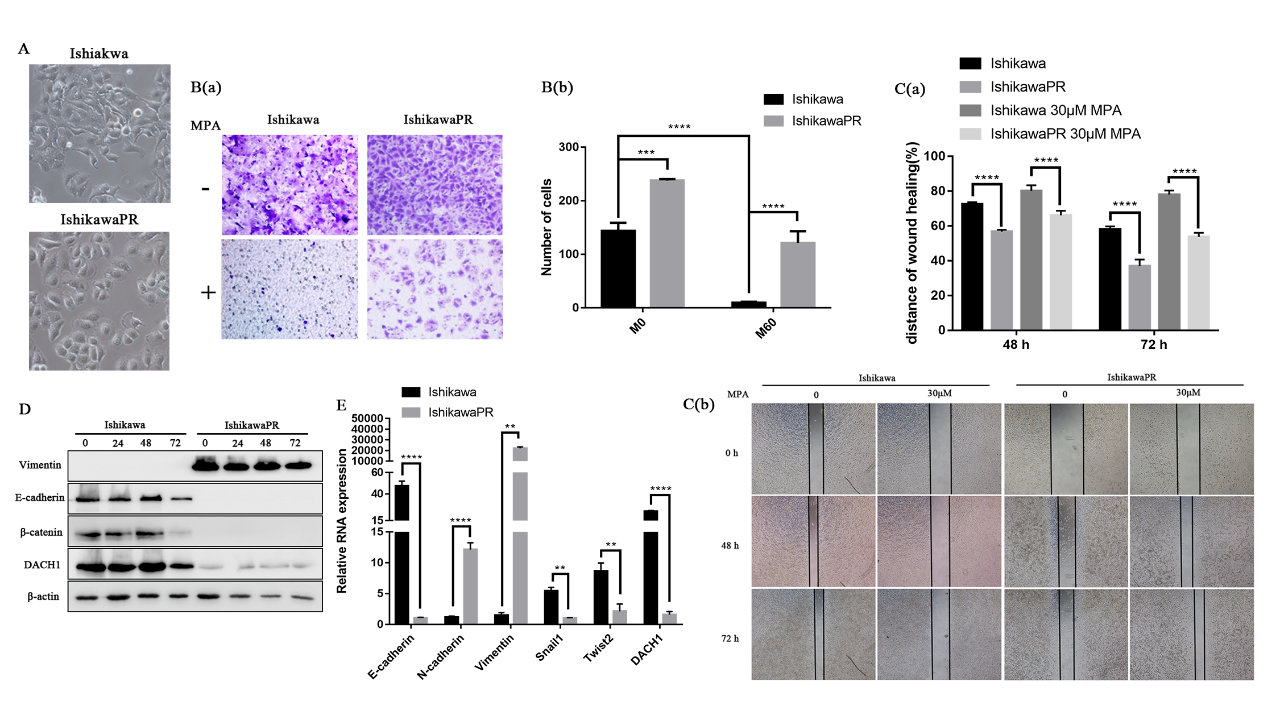
**

**Supplementary Figure 1.** (**A**) Phase-contrast microscopic images of Ishikawa and IshikawaPR cell. (**B**) Transwell migration assay evaluated the motility ability of Ishikawa and IshikawaPR cell with or without MPA treatment. (**C**) Representative wound healing at 0,48,72 hours after treated with 0,30 μM MPA respectively for 48h. (**D**) Western blot of DACH1 and EMT markers after treatment with 15 μM MPA for 0,24,48,72h. (**E**) PCR result of EMT markers of Ishikawa and IshikawaPR cells.
